# First report of *Rhodnius montenegrensis* (Hemiptera: Reduviidae: Triatominae) in Bolivia

**DOI:** 10.1590/0037-8682-0156-2022

**Published:** 2022-10-24

**Authors:** André Luiz Rodrigues Menezes, Ricardo Angelo Schneider, Mariane Albuquerque Lima Ribeiro, Cícera Alexsandra Costa dos Santos, Elaine Oliveira Costa de Carvalho, Gabriel Cestari Vilardi, Jader de Oliveira, João Aristeu da Rosa

**Affiliations:** 1 Instituto Federal de Educação, Ciências e Tecnologia de Rondônia, Campus Guajará-Mirim, Guajará-Mirim, RO, Brasil.; 2 Universidade Estadual Paulista Júlio de Mesquita Filho, Programa de Pós-Graduação Stricto Sensu em Biociências e Biotecnologia, Araraquara, SP, Brasil.; 3 Universidad Autónoma del Beni “José Ballivián” - UAB, Guayaramerín, Beni, Bolivia.; 4 Universidade Federal do Acre, Centro de Ciências da Saúde e do Desporto, UFAC, Rio Branco, AC, Brasil.; 5 Fundação Universidade Federal de Rondônia, Departamento Acadêmico de Ciências Sociais e Ambientais, Campus de Guajará-Mirim, Guajará-Mirim, RO, Brasil.; 6 Universidade de São Paulo, Faculdade de Saúde Pública, Laboratório de Entomologia em Saúde Pública, São Paulo, SP, Brasil.; 7 Universidade Estadual Paulista Júlio de Mesquita Filho, Faculdade de Ciências Farmacêuticas, Araraquara, SP, Brasil.

**Keywords:** Entomological surveillance, Western Amazon, Kissing bugs, New record

## Abstract

**Background:**

The subfamily Triatominae, which comprises 157 species, carries the protozoan *Trypanosoma cruzi*, the etiological agent of Chagas disease. This short communication reports for the first time the occurrence of *Rhodnius montenegrensis* in Bolivia.

**Methods:**

Active searches were carried out on palm trees of the genus *Oenocarpus* in Beni district, Bolivia.

**Results:**

Fifteen *R. montenegrensis* specimens were collected from a rural area of the Beni district, Bolivia, and tested positive for *T. cruzi*.

**Conclusions:**

This new report expands the geographic distribution of the species in Latin America. Due to their ability to transmit trypanosomatids, the species deserves the attention of vector control programs.

The subfamily Triatominae is currently composed of 157 species with 18 genera^1^ ^-^ ^3^. In Bolivia, there are 20 species of triatomines: *Eratyrus mucronatus* Stål, 1859; *Microtriatoma trinidadensis* (Lent, 1951); *Panstrongylus geniculatus* (Latreille, 1811); *Panstrongylus guentheri* Berg, 1879; *Panstrongylus megistus* (Burmeister, 1835); *Panstrongylus noireaui* Gil-Santana et al., 2022; *Panstrongylus rufotuberculatus* (Champion, 1899); *Panstrongylus diasi* Pinto & Lent, 1946; *Psammolestes coreodes* Bergroth, 1911;; *Rhodnius micki* Zhao et al., 2021; *Rhodnius prolixus* Stål, 1859; *Rhodnius robustus* Larrousse, 1927; *Rhodnius stali* Lent et al. 1993; *Triatoma boliviana* Martínez et al., 2007; *Triatoma delpontei* Romaña & Abalos, 1947; *Triatoma garciabesi* Carcavallo et al., 1967; *Triatoma guasayana* Wygodzinsky & Abalos, 1949; *Triatoma infestans* (Klug, 1834); *Triatoma sordida* Stål, 1859; and *Triatoma venosa* Stål, 1872^4^ ^-^ ^6^. 

*Rhodnius Stål*, 1859 species are present in wild areas and consequently maintain the enzootic cycle, with the main ecotopes being palm trees of the genus *Attalea*. However, they have been reported to be present in and around homes (intradomiciliary and peridomiciliary), posing an epidemiological risk of transmission of Chagas disease, since they act as significant transmitters of natural infection with *Trypanosoma cruzi* (Chagas, 1909) (Kinetoplastida: Trypanosomatidae)^7^ ^-^ ^8^. 

The species *Rhodnius montenegrensis* has been registered only in Brazil, occurring in the states of Acre^9^, Amazonas^10^, Rondônia^11^, and Roraima^12^. This distribution has been expanded with the present study, which aims to describe the first report of the occurrence of *R. montenegrensis* in Bolivia. Between 2019 and 2020, 15 specimens of *R*. *montenegrensis* were collected from rural areas in the municipality of Guayaramerin, Beni, and Bolivia (Figure 1).

**FIGURE 1:**
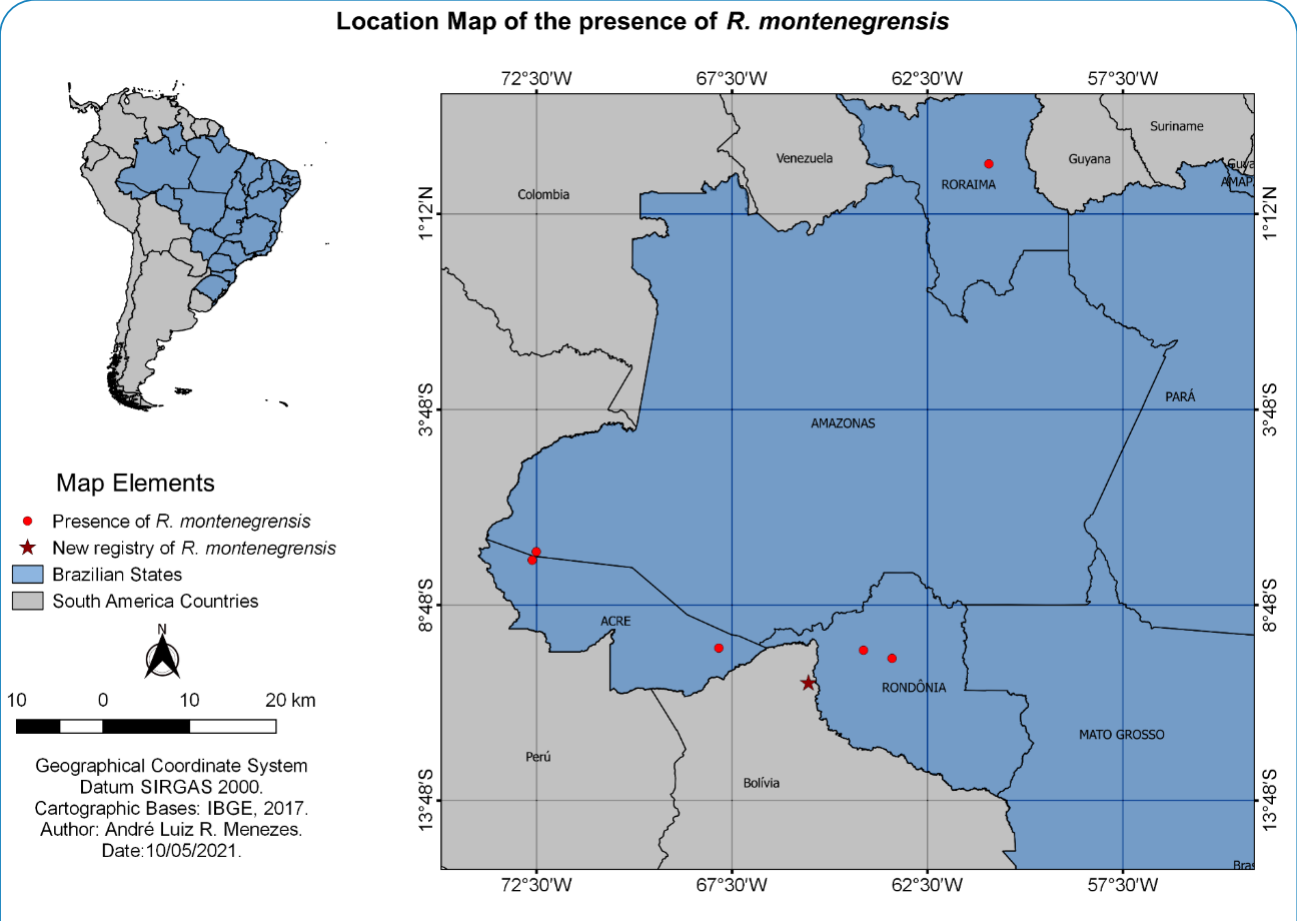
Location of *R. montenegrensis* collection in Bolivia in 2019.

Guajará-Mirim, in Brazil, borders Guayaramerin, located in Bolivia, and is considered a twin city separated by the Mamoré River^13^. The first record of the species *R. montenegrensis* in 2012 was made in the municipality of Monte Negro^11^, Rondônia, a city close to Guajará-Mirim; thus, we may justify the presence of this specimen in Bolivia due to the surroundings of these municipalities.

The specimens were collected by means of an active search in palm trees (genus *Oenocarpus*) distributed in areas of secondary forests and pasture lands. Using tweezers and machetes to assist in thinning, the bracts were removed (as they can lodge a large number of invertebrates and small vertebrates), and the triatomines were collected one at a time to avoid damage. We captured 15 triatomines - nine adults, three fourth-instar nymphs, and three fifth-instar nymphs of *R. montenegrensis*. 

For identification, we used the method described by Rosa et al. (2012)^11^. Fifteen triatomines were subjected to infection analysis and identification at the Parasitology Laboratory of the Department of Biological Sciences, Faculty of Pharmaceutical Sciences, Universidade Estadual Paulista, Campus de Araraquara. The specimens were added to the Triatominae collection (Figure 2) by Dr. José Maria Soares Barata (CTJMSB) at UNESP, Araraquara. The infectious capability of the triatomines was investigated by molecular analysis [conventional polymerase chain reaction (PCR)], to detect and confirm the presence of *T. cruzi*, the etiologic agent of Chagas disease.

**FIGURE 2:**
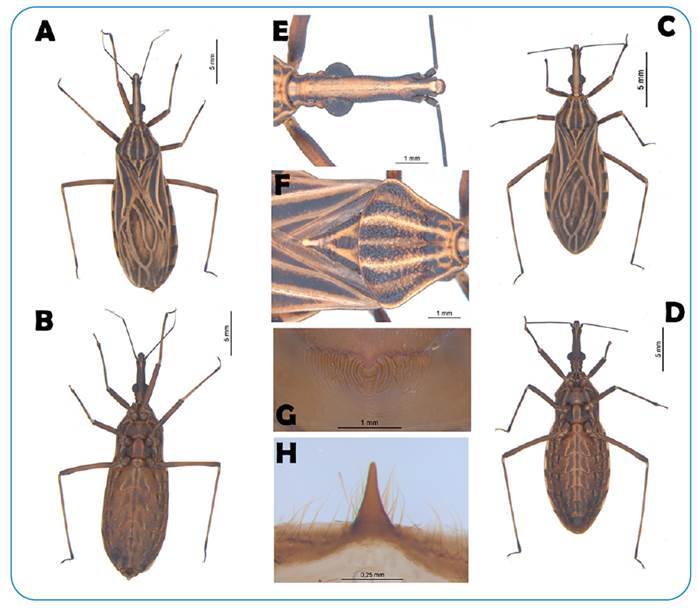
*Rhodnius montenegrensis* specimen captured in a palm tree in the municipality of Guayaramerin, Beni, Bolivia. Legends: *R. montenegrensis*. **A:** female: dorsal side; **B:** female: ventral side; **C:** male: dorsal side; **D:** male: ventral side; **E:** head; **F:** pronotum and scutellum; **G:** process of the I urotergite; **H:** male external genitalia - median process of the pygophore.

To identify *T. cruzi*, we first extracted the DNA with the PureLink™ Genomic DNA Mini Kit (Thermo Fisher Scientific, MA, USA), using the digestive tract of each collected triatomine suspended in absolute alcohol and stored at -20°C. For the PCR, we followed the kDNA-PCR protocol described by Márquez et al. (2016)^14^. Among the 15 samples tested, 6 yielded positive results for *T. cruzi* (Figure 3).

**FIGURE 3:**
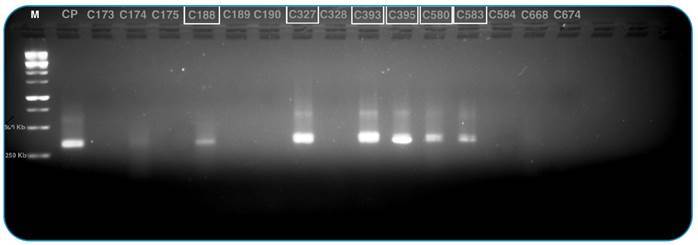
Caption: M - Marker (Ladder 1kb), CP - positive control (Y strain), Samples - C188, C327, C393, C580, C583 tested positive for *T. cruzi*. We used the forward primer 121: 5′ · AAATAATG TACGGGKGAGATGCATGA · 3′ and reverse primer 122: 5′ · GGTTCGATT GGGGTTGGTGTAATATA · 3′.

*Rhodnius montenegrensis* and *R. robustus* present some morphological similarities; however, morphological^15^, morphometric^15^, transcriptomic^15^, and cytogenetic^16^ studies have allowed differentiation of the species and confirmed the specific status of *R. montenegrensis*. For this reason, there may be an erroneous description of the distribution of *R. montenegrensis*, both in Brazil and in other Latin American countries (with an emphasis on countries bordering Brazil).

*R. montenegrensis* specimens collected from palm trees and residences revealed their ability to adapt to the human environment, dispersal, and mobility^17^. Studies conducted in Acre, Amazonas, and Rondônia demonstrate the predominance of this species in its natural ecotope, and intrusion into residences and the infection rate for *T. cruzi* in this species are significant in the localities where they were captured^9^ ^-^ ^11^ ^,^ ^17^. Another aspect described in these studies is the non-occurrence of domiciliation of *R. montenegrensis* ^10^ ^,^ ^17^. 

With the expansion of this species in Brazilian states and a neighboring country, such as Bolivia, it is pertinent to affirm the epidemiological importance of including this species in the transmission cycle of Chagas disease in the Brazilian and international Amazon.

*Rhodnius montenegrensis* has also been reported in domestic environments, but only in the countryside^18^. In addition, it has been found to be naturally infected with *Trypanosoma rangeli* Tejera, 1920, which is of major importance because the difficulty in isolation and diagnosis may be related to a double trypanosomatid infection, which can lead to false positive or true positive results for Chagas disease^18^ ^,^ ^19^. This new report on the occurrence of *R. montenegrensis* expands the geographic distribution of the species in Latin America, with Bolivia being the second country to register the presence of the insect and increasing the number of species described in the locality. 
